# Silicon-Based Polymer-Derived Ceramics as Anode Materials in Lithium-Ion Batteries

**DOI:** 10.3390/ma18153648

**Published:** 2025-08-03

**Authors:** Liang Zhang, Han Fei, Chenghuan Wang, Hao Ma, Xuan Li, Pengjie Gao, Qingbo Wen, Shasha Tao, Xiang Xiong

**Affiliations:** 1 State Key Laboratory of Powder Metallurgy, Central South University, Changsha 410083, China; 2 School of Materials Science and Engineering, Central South University, Changsha 410083, China

**Keywords:** polymer-derived ceramics, lithium-ion battery, anode materials, free carbon

## Abstract

In most commercial lithium-ion batteries, graphite remains the primary anode material. However, its theoretical specific capacity is only 372 mAh∙g^−1^, which falls short of meeting the demands of high-performance electronic devices. Silicon anodes, despite boasting an ultra-high theoretical specific capacity of 4200 mAh∙g^−1^, suffer from significant volume expansion (>300%) during cycling, leading to severe capacity fade and limiting their commercial viability. Currently, silicon-based polymer-derived ceramics have emerged as a highly promising next-generation anode material for lithium-ion batteries, thanks to their unique nano-cluster structure, tunable composition, and low volume expansion characteristics. The maximum capacity of the ceramics can exceed 1000 mAh∙g^−1^, and their unique synthesis routes enable customization to align with diverse electrochemical application requirements. In this paper, we present the progress of silicon oxycarbide (SiOC), silicon carbonitride (SiCN), silicon boron carbonitride (SiBCN) and silicon oxycarbonitride (SiOCN) in the field of LIBs, including their synthesis, structural characteristics and electrochemical properties, etc. The mechanisms of lithium-ion storage in the Si-based anode materials are summarized as well, including the key role of free carbon in these materials.

## 1. Introduction

### 1.1. Anode Materials in Lithium-Ion Batteries

**Figure 1:**
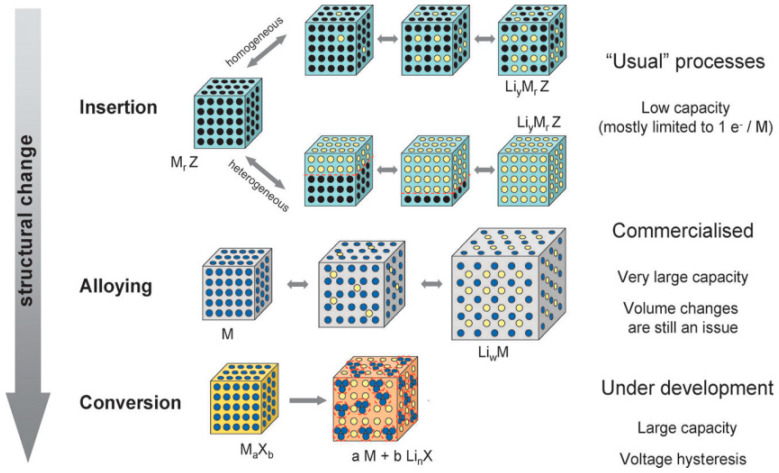
A schematic representation of the different reaction mechanisms observed in electrode materials for lithium batteries. Black circles: voids in the crystal structure, blue circles: metal, yellow circles: lithium. Copied with permission from the Royal Society of Chemistry [25].

From portable electronic devices to electric vehicles and even to military and space applications, lithium-ion batteries (LIBs) are widely used in all fields of our daily life because of its high voltage, high energy density, low self-discharge and no memory effect. Lithium-ion batteries are mainly composed of the cathode electrode (including the cathode collector fluid, such as metal aluminum), the anode electrode (including the anode collector fluid, such as metal copper), the electrolyte and the diaphragm [1,2]. In any type of lithium-ion battery, the properties of the anode material are of great significance to the performance of the whole lithium-ion battery [3,4]. Ideally, a suitable anode material should meet the following conditions: (1) a low lithium extraction/insertion potential, allowing for a significant voltage difference with the cathode material, thereby resulting in a high-voltage battery; (2) a high reversible specific capacity, which enhances the overall battery capacity; (3) the easy insertion and extraction of lithium ions, ensuring high coulombic efficiency; (4) good electronic conductivity and ionic conductivity; (5) excellent stability and compatibility with the electrolyte; and (6) abundant raw material sources, being safe and environmentally friendly. So far, various materials have been developed as anode materials for LIBs, based on three types of storage mechanisms (Figure 1): (1) insertion; (2) alloying; and (3) conversion. For instance, Sn-based electrodes, Si-based electrodes, Ge-based electrodes, metal nitride-based electrodes, and metal oxide-based electrodes follow either alloying or conversion mechanisms [5]. Carbonaceous materials (e.g., graphite) and Li_4_Ti_5_O_12_ (LTO) are based on insertion mechanisms [6]. Among various anode materials, graphite remains one of the most widely used anode materials, because it has excellent mechanical thermal stability, good electrical conductivity, low working voltage, non-toxicity, abundant storage, and it can avoid the dendrites formed in the charging and discharging process [7,8]. However, graphite has a low theoretical capacity of 372 mAh∙g^−1^, which cannot meet the demand for high energy density batteries. In addition, due to the large distance between the graphite layers, the electrolysis solution may also be separated from lithium embedding, causing the layered structure to collapse, leading to structural destruction which in turn affects cycle life [9]. In these anode materials, Si has the highest theoretical specific capacity (4200 mAh∙g^−1^) which can reach more than ten times that of traditional graphite. This gives an Si anode enormous application potential [10,11]. Since the lithiation and delithiation of silicon are alloying reactions (Figure 1), they involve significant volume expansion and shrinkage due to the alloy density changes, with a rate of change exceeding 300% and brings irreversible effects on the electrochemical properties, including the reduction of cycle life and the decrease of coulomb efficiency [12]. The resulting internal stress seriously affects the stability of the structure, not only in the rupture of the structure of the material itself, also reflected in the material macroscope separation from the fluid collection, seriously influencing the cycle stability. Also, broken silicon in turn brings the internal silicon in contact with the electrolyte, continuous generation of new SEI (solid electrolyte interface), and this leads to a constant reduction of the active material. This is the main reason for the low efficiency of the first circle of the silicon anode materials [13]. To alleviate the significant volume expansion of Si anode materials during cycling, researchers have primarily proposed three solutions: (1) the nano-structuring of Si [14]; (2) composite integration with materials that have pressure-buffering properties [15]; and (3) designing physical barriers to provide space for the expansion of Si [16]. However, these methods generally lead to capacity degradation.

Since the middle of 1990s, Si-based polymer-derived ceramics have been considered as anode materials of lithium ion batteries in some patent applications [17,18]. In the last two decades, this research topic has attracted increasing attention because the Si-based ceramics (e.g., SiOC, SiCN) have been proven to exhibit excellent electrochemical properties superior than or unobtainable for other materials [19,20,21]. Compared to silicon, the silicon-based PDCs demonstrate enhanced structural stability, minimal volumetric expansion, straightforward fabrication processes, and high compatibility with large-scale manufacturing requirements [22,23,24]. Therefore, in this work, we pay more attention to polymer-derived ceramics.

### 1.2. Si-Based Polymer-Derived Ceramics

**Figure 2:**
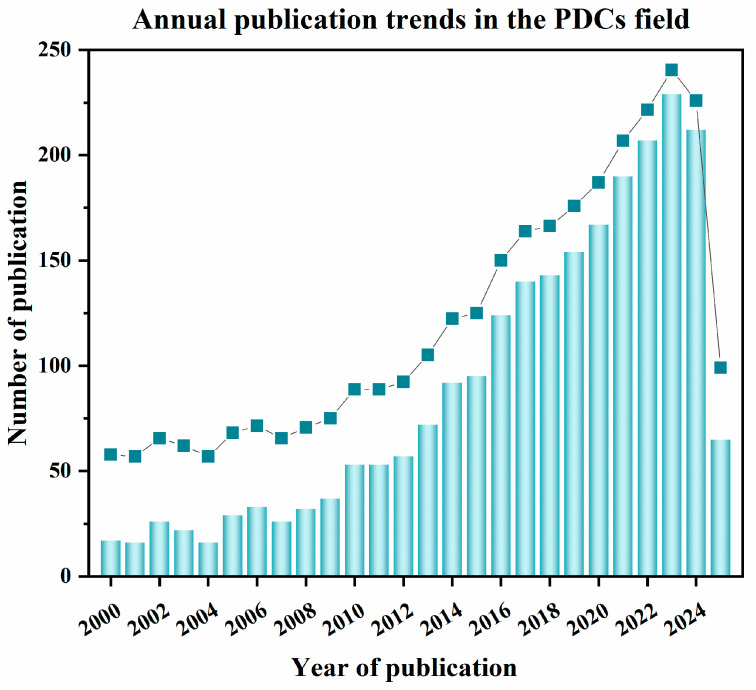
Number of publications resulting from a search with the key word “polymer derived ceramic”, from 2000 up to 10 May 2025 (data from Web of Science).

Si-based polymer-derived ceramics is a new category of advanced ceramics produced via pyrolysis of organosilicon polymers which has been developed in the early 1960s. Owing to structural and functional superiorities as well as their ability for being shaped by various processing techniques, people gradually pay more attention to the progress of polymer−derived ceramics. The number of publications resulting from a search with the key word “polymer derived ceramic” from 1 January 2000 to 10 May 2025 is shown in Figure 2. The most known classes of PDCs are in the binary systems Si_3_N_4_, SiC, BN, and AlN, ternary systems SiCN, SiCO, and BCN as well as in the quaternary systems SiCNO, SiBCN, SiBCO, SiAlCN, and SiAlCO. In recent years, several pentanary systems of PDCs (e.g., SiHfBCN, SiHfCNO) have been reported as well [18,26].

**Figure 3:**
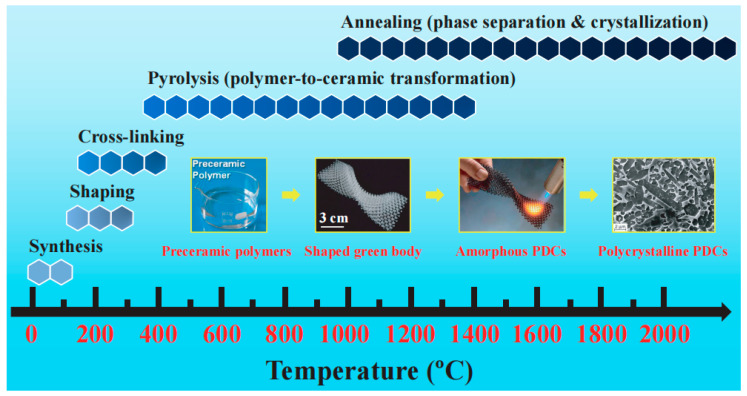
The process of converting polymers into ceramics. Copied with permission from Elsevier [26].

Polymer derived Si-based ceramics exhibit several beneficial properties for application as anode materials in lithium-ion batteries. They are chemically inert with respect to battery components and are lightweight materials. In addition, they are able to minimize the agglomeration of lithium ions. Importantly, the chemical and physical properties of the PDCs can be tailored via tuning the molecular structure of starting polymers and/or controlling the thermal treatment parameters. The preparation of silicon-based polymer-derived ceramics (PDCs) typically involves three steps: (1) synthesis of Si-containing preceramic polymers; (2) shaping and crosslinking (100–400 °C); (3) polymer-to-ceramic transformation (400–1400 °C). After polymer-to-ceramic conversion, the PDCs are generally amorphous (Figure 3) [18]. Upon annealing at higher temperatures (≥1400 °C), they will transform into (poly)crystalline ceramics [27]. Two typical polymer-derived ceramics are silicon oxycarbide (SiOC) and silicon carbonitride (SiCN), both of which possess unique microstructures not commonly found in other ceramic materials. SiOC is typically amorphous, with Si atoms tetrahedrally coordinated by O and C atoms. The microstructure of amorphous SiCN ceramics is primarily influenced by the molecular structures of the preceramic polymers. The key difference between amorphous SiCN ceramics is whether there are Si–C/N mixed bonds (the presence of SiCxNy units) in the ceramic matrix or not [27]. Due to their physical–chemical and functional properties and their capabilities of being shaped using lots of processing ways, PDCs have the broad application potential in the field of lithium-ion batteries. However, PDCs remain far from practical applications due to persistent challenges such as low initial coulombic efficiency and an incompletely understood lithium storage mechanism. To address this knowledge gap, this review systematically examines recent advances in SiOC, SiCN, SiBCN and SiOCN for lithium-ion battery applications, with a focus on their synthesis protocols, structural characteristics, and electrochemical performance. Furthermore, we elucidate the lithium storage mechanisms in PDCs, highlighting the critical role of free carbon phases in enhancing their electrochemical behavior.

## 2. SiOC-Based Anode Materials

SiOC is a kind of silicon-based anode material, which is an amorphous ceramic material composed of Si, O and C elements bonded to each other, generally through the precursor conversion method. The silicon-containing polymer (polysiloxane, polycarbosilane and sesquisilane, etc.) is transformed into amorphous SiOC ceramic material after cross-linking and subsequent pyrolysis [28,29]. Dahn et al. [30] studied the pyrolysis behavior of PMPS and PPSSO and characterized the products. They found that the pyrolysis products are amorphous structures and can reversibly intercalate lithium. Interestingly, the ceramics can achieve good reversible capacity at relatively low voltages, which opened the boom of research on SiOC anode materials [13].

**Figure 4:**
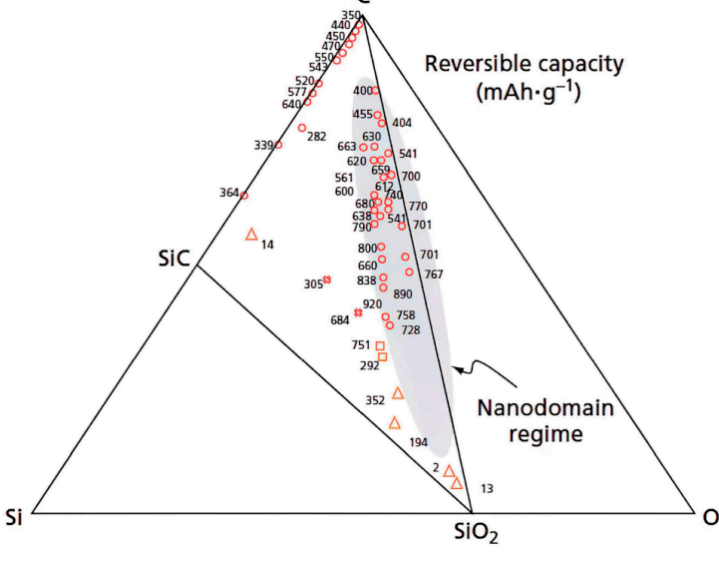
SiOC compositional triangle. The numbers reflect composition-dependent reversible capacity in SiOC anodes, with maxima occurring in mixed-bond regimes (grey region) characterized by tetrahedral Si-O-C coordination Copied with permission from Springer Nature [27].

Furthermore, Dahn et al. [30] discovered reversible lithium-ion intercalation in SiOC materials at potentials below 1 V, achieving a specific capacity of near 600 mAh·g^−1^. By utilizing siloxane units with varying functional groups and employing diverse synthesis methods, the team synthesized over 60 SiOC anode variants (Figure 4). The numbers represent the reversible capacity of the SiOC anode material as a function of its composition. The highest capacity values are observed in compositions that contain mixed Si bonds (grey area), where silicon is tetrahedrally bonded to both oxygen and carbon [3].

**Figure 5:**
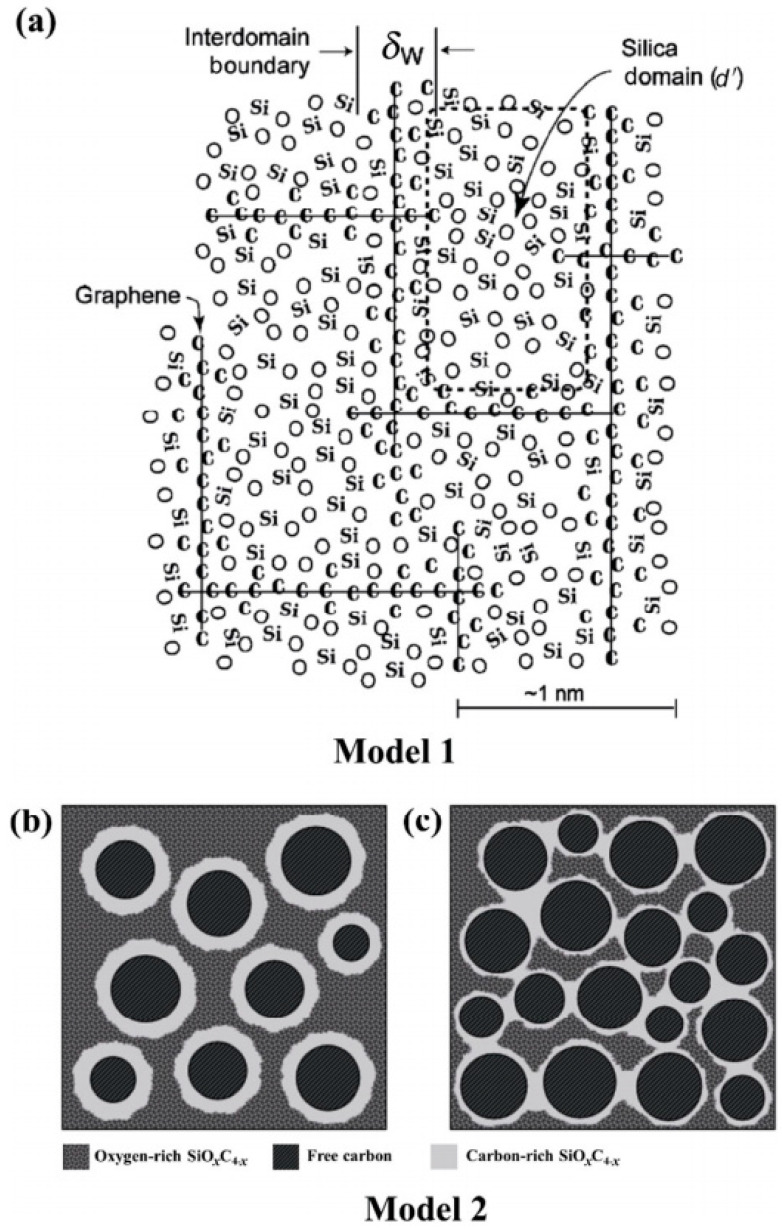
Two proposed models illustrating the microstructure of polymer-derived SiOC ceramics. Copied with permission from Springer Nature [27]. (**a**) a classic model featuring a microstructure with clusters of silica tetrahedra, a monolayer of SiC_x_O_4−x_ mixed bonds, and a graphene cage-like network that encapsulates the silica nanodomains; (**b**) Model 2 with an isolated carbon-rich SiO_x_C_4−x_ interface; and (**c**) Model 2 with an interconnected carbon-rich SiO_x_C_4−x_ interface in carbon-rich SiOC ceramics [2].

Two proposed models illustrate the microstructure of polymer-derived SiOC ceramics (Figure 5).

Monomers of commercially available polysiloxanes and organosilicon polymers can be used as precursors, both of which contain Si-O-Si bonds in their molecular structures, and the groups of the side chains can be volatilized during pyrolysis, which facilitates the introduction of C atoms into the side chains. SiOC is composed of an amorphous SiO_x_C_(4−x)_ (0 < x ≤ 4) glass phase and a free carbon phase. The glass phase contains four phase structures: SiO_3_C, SiO_2_C_2_, SiOC_3_ and SiO_4_, which are formed by different numbers of C atoms replacing the O atoms in the SiO_2_ tetrahedral structure, in which the C atoms are sp^3^ hybridized. Free carbon, on the other hand, is free in the whole phase structure in the form of sp^2^ hybridization, and is used as a framework to support the whole structure [13].

In recent research, V.S. Pradeepa et al. [31] prepared SiOC materials by pyrolysis using pre-ceramic polymers and crosslinkers such as 1,3,5,7-tetramethyl1,3,5,7-tetravinylcyclotetrasiloxane or divinylbenzene, and the obtained SiOC anode showed a lithium storage capacity of up to about 1300 mAh∙g^−1^, but there was an irreversible capacity loss during the first charge and discharge. After optimizing the balance between silicon-oxycarbon-silicon and free-carbon phases, the high-carbon SiOC material has a measured reversible capacity of 650 mAh∙g^−1^ and exhibits excellent cycling stability and high charge/discharge rate performance, with a reversible capacity of approximately 200 mAh∙g^−1^ at 2C. Their study suggests that the high capacity of the SiOC anode may be related to the complex amorphous silicon-oxy-carbon structure, in which the silicon atoms share bonds with both oxygen and carbon atoms. Possible reversible lithium storage locations include hybrid Si-O-C tetrahedra forming an amorphous network, micropores of the glass phase, and free carbon clusters.

In order to achieve high capacity retention in SiOC materials, Dragoljub Vrankovic et al. [19] proposed a cost-effective synthetic route in which silicon-based composites are encapsulated from highly porous silicon (obtained through silicate reduction) in an organic carbon and polymer-derived silicon-oxycarbon (C/SiOC) matrix. This method yields silicon-based anode materials with a capacity of 2000 to 3000 mAh∙g^−1^, a coulombic efficiency of over 99.5%, and nearly 100% capacity retention over more than 100 cycles. Molecular dynamics simulations revealed that the highly porous silicon morphology provides a strained free volume, thus preventing macroscopic changes during the initial Li-Si alloying process. The carbon layer ensures electrical contact, while the SiOC matrix significantly reduces the interface between the electrolyte and the electrode material, thereby inhibiting the formation of a solid electrolyte interphase on the silicon.

Different atmospheres will influence the structure of SiOC. Magdalena Graczyk-Zajac et al. [32] compared two SiOC glasses synthesized by pyrolysis in different atmospheres, one in argon and the other in carbon dioxide. The argon pyrolysis of SiOC (SiOC-Ar) exhibits a higher reversible lithium-ion storage capacity (325 mAh∙g^−1^), while the CO_2_ pyrolysis of SiOC (SiOC-CO_2_) is 165 mAh∙g^−1^. NMR and spectroscopy analysis showed that the atmosphere affected the material structure, CO_2_ led to the conversion of SiOC to silica network, and there may be more activated carbon in the Ar atmosphere. UV-Raman revealed that there were more defective carbon phases in Ar-treated aerogels, and more ordered carbon phases in CO_2_-treated aerogels, which affected the lithium-ion storage capacity. Therefore, it can be seen that the more disordered carbon phase in SiOC-Ar is conducive to lithium-ion storage, and this disorder is caused by the hybrid bonding unit.

**Figure 6:**
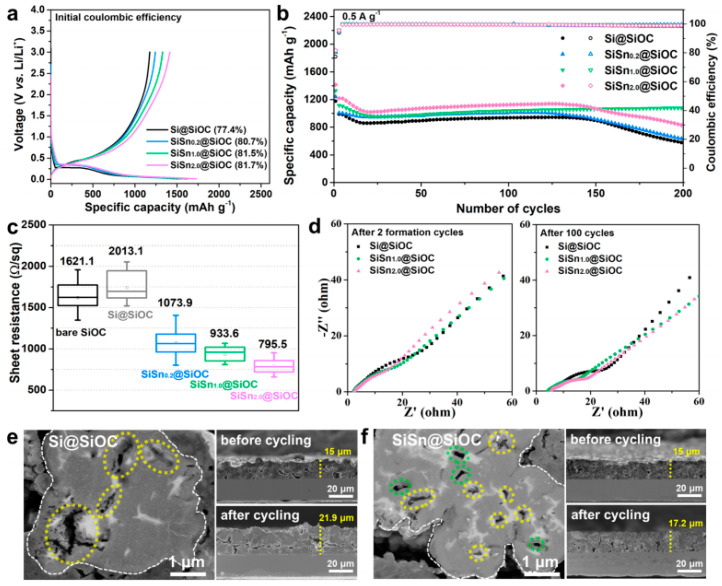
(**a**) Initial GCD curves and (**b**) cycling stability of Si@SiOC and various SiSnx@SiOC electrodes. (**c**) Sheet resistances of bare SiOC, Si@SiOC, and various SiSnx@SiOC electrodes. (**d**) Nyquist plots of Si@SiOC and SiSn@SiOC electrodes after formation cycles and 100 cycles. Cross-sectional SEM images after 100 cycles and thickness change before and after cycling of (**e**) Si@SiOC and (**f**) SiSn@SiOC electrodes (delithiated states) Copied with permission from the Royal Society of Chemistry [33].

Although SiOC anodes have many advantages relative to graphite and silicon, the practical application of SiOC as an anode material is hindered by its inherent characteristics. Its capacity has currently reached a bottleneck and requires further improvement, while issues such as poor conductivity and low initial coulombic efficiency need to be addressed. By combining SiOC with other materials, such as high-capacity tin or silicon nanoparticles [33], carbon nanotubes [34], graphene [24,35], and reduced graphene oxide, and designing its structure to leverage the advantages of each, multiple challenges can be solved simultaneously. For example, G. Blugan et al. [36] investigated SiOC/Sn nanocomposites, where disordered carbon serves as an effective charge carrier and an active site for lithium-ion insertion, thereby enhancing electrochemical performance. The SiOC/Sn nanocomposites were synthesized using functional PEOS siloxanes. This approach helps prevent particle crushing and cracking at the rough solid/electrolyte interface, promoting a uniform distribution of metal nanoparticles within the PDCs matrix through carbon-thermal reduction. By inhibiting the aggregation of Sn in the SiOC matrix, a uniform lithium-ion diffusion path is maintained, which enhances both electrochemical performance and cycle life. Additionally, the high-rate performance of SiOC/Sn nanocomposites facilitates fast lithium-ion diffusion kinetics and efficient electron transport. The specific capacities of these nanocomposites range from 200 to 1300 mAh∙g^−1^ and improve cycling stability and lithium-ion diffusion efficiency by Li_22_Sn_5_ reversible formation of alloys during lithiation/delithiation. These mechanisms make SiOC/Sn nanocomposites exhibit excellent electrochemical properties as anode materials for lithium-ion batteries, which is expected to solve the volume expansion problem of alloy materials and improve the energy density of batteries. Furthermore, Kwanghyun Do et al. [33] enhances Si anodes via a dual-matrix design. Covalent seeding embeds Si nanoparticles within a SiOC buffer (Si@SiOC), showing an improved capacity. Adding tin nanocrystals (SiSn@SiOC) creates internal voids to accommodate volume changes and boosts conductivity. The optimized composite delivers 1330 mAh∙g^−1^ capacity and exceptional cycling stability (96.5% retention after 200 cycles). The relevant performance is shown in the Figure 6. It validates the dual-matrix design as a holistic solution for Si anodes: Sn simultaneously enhances capacity (alloying), conductivity (metallic pathways), and cyclability (void-mediated stress relief). The optimal SiSn_1.0_@SiOC formulation balances high energy density (1329 mAh∙g^−1^) with unprecedented mechanical–electrochemical durability, setting a benchmark for structurally integrated composites.

**Figure 7:**
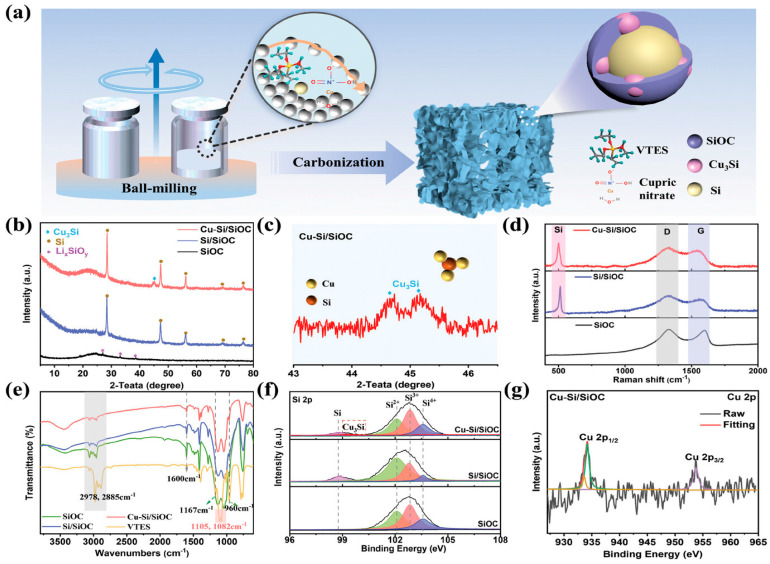
(**a**) Synthesis and structure diagram of Cu-Si/SiOC. (**b**) XRD patterns of SiOC-based samples. (**c**) XRD pattern of Cu-Si/SiOC. (**d**) Raman spectra, (**e**) FTIR spectra, and (**f**) high-resolution Si 2p spectra of SiOC-based samples. (**g**) High-resolution Cu 2p spectrum of Cu-Si/SiOC. Copied with permission from John Wiley [37].

Ahn et al. [33] employed covalent-assisted seeding technology to establish robust covalent bonds between silicon nanoparticles and the SiOC matrix. They further introduced a dual-matrix design by incorporating tin nanocrystals as pore triggers within the Si@SiOC framework, thereby creating internal artificial voids. The resulting SiSn@SiOC composite demonstrated excellent electrochemical performance. Similarly, Wang et al. [37] recently engineered a 3D micron-sized SiOC anode with an in situ Cu_3_Si interphase through solvent-free ball milling (Figure 7). The conductive Cu_3_Si alloy buffered mechanical stress, enhanced interfacial stability, and facilitated an LiF-rich SEI, achieving 78% initial coulombic efficiency and remarkable long-term cycling (750 mAh∙g^−1^ after 1000 cycles at 1 A∙g^−1^) in half-cells, alongside 93% capacity retention in NCM811 full cells. Ma et al. [35] developed a three-dimensional (3D) lamellar SiOC@C/rGO composite through a hydrothermal reaction and electrostatic self-assembly process. In this composite, SiOC powders are encapsulated by amorphous carbon layers and uniformly dispersed within reduced graphene oxide (rGO) sheets. The combination of carbon-free nanoclusters in SiOC, carbon layers on its surface, and the rGO support creates a multidimensional interconnected conductive network, improving interfacial adhesion. As a result, the SiOC@C/rGO composite exhibits a high specific capacity (676 mAh∙g^−1^ at 200 mA g^−1^) and exceptional rate capability (306.4 mAh∙g^−1^ at 4000 mA g^−1^). When integrated into a full cell with a LiFePO_4_ cathode, it shows a stable voltage platform and reliable performance over 200 cycles. The superior electrochemical performance of SiOC@C/rGO is attributed to the synergistic effects of its robust structure, multidimensional conductive network, and enhanced chemical stability. Li et al. [38] use in situ Raman spectroscopy revealed that the vertically grown graphene coating on the Hp-SiOC@VG anode primarily enhances conductivity rather than contributing significantly to Li^+^ storage. In addition, by analyzing the changes in the position and intensity of the G-band, the reversibility of the graphene layers during the lithium insertion and extraction processes was revealed. These results suggest that vertical graphene is not the primary contributor to lithium storage but instead enhances the electrochemical performance of the electrode by providing a conductive network. Furthermore, the issue of low initial coulombic efficiency of SiOC anodes can be overcome using prelithiation methods. Lin et al. [39] employed an in situ chemical pre-lithiation strategy to synthesize onion-like SiOC/C spheres with high initial coulombic efficiency. This was achieved through one-step injection pyrolysis of lithium hepta(i-butyl) silsesquioxane trisilanolate. During the pyrolysis process, Li_x_SiO_y_ is uniformly formed within the onion-like SiOC/C spheres, which significantly minimizes the irreversible consumption of Li ions. This also shows a high initial coulombic efficiency of ca. 80% and good cycling stability after 500 cycles. This demonstrates the promising potential of the in-situ chemical pre-lithiation strategy for applications in lithium-ion batteries.

## 3. SiCN-Based Anode Materials

**Figure 8:**
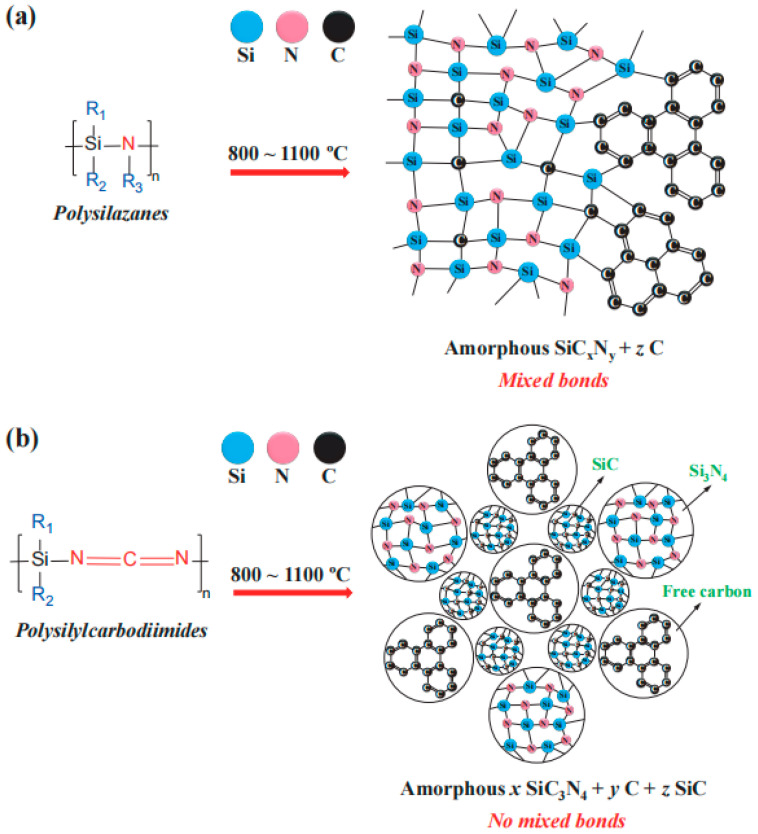
Schematic illustration of the microstructure of amorphous SiCN ceramics derived from polysilazanes (**a**) and from polysilylcarbodiimides (**b**). Copied with permission from Springer Nature [27].

The SiCN is obtained by pyrolysis of polysilazane precursors. Diverging from SiOC systems, amorphous silicon carbonitride (SiCN) ceramics exhibit precursor-dependent microstructural configurations that may lack defined SiC_x_N_y_ moieties (featuring Si–C/N bonding). Structural analysis in Figure 8 illustrates this dichotomy: polysilazane-derived SiCN contains tetrahedral Si centers coordinated exclusively with N, C, or their hybrid configurations (SiC_4_, SiN_4_, or SiC_x_N_y_), while counterparts synthesized from polysilocarbodiimide manifest a triphasic amorphous architecture comprising Si_3_N_4_, SiC, and free carbon nanodomains, with complete absence of SiO_4−x_C_x_ units.

SiCN material has a robust three-dimensional network structure with chemical resistance, thermal stability and oxidation resistance [40,41]. It can ensure the stability of the electrode material structure during charging and discharging processes, thereby improving the performance of batteries. Its research as an anode material has become one of the hotspots in lithium-ion batteries [42]. In 1997, Dahn et al. [30] first prepared SiCN ceramics using thermal decomposition of polysilazane and used it as a anode material for lithium-ion batteries. The study found that its reversible capacity can reach up to 560 mAh∙g^−1^. Upon discovering that SiCN material possesses high charge and discharge capacities and exceptional properties, Feng’s team identified two structural advantages that render it suitable as an active material for lithium-ion battery anodes [43]. Firstly, the SiCN network contains numerous nanoclusters and free dangling bonds between silicon and carbon, serving as active sites for lithium-ion intercalation/de-intercalation and boasting high electrochemical capacity. Secondly, the nanoholes or nanochannels within the SiCN network offer numerous smooth channels for lithium-ion transport, thereby facilitating superior electrochemical kinetic performance. Consequently, they employed 1000 °C argon gas heat treatment to fabricate SiCN-CNTs (10 wt% CNTs) composites. Charge and discharge cycling tests revealed that their initial specific discharge capacity was 1.5 times that of pure SiCN, signifying a notable enhancement and improving the material’s conductivity.

Contemporary investigations into silicon carbonitride (SiCN) anodes fabricated via polymer-derived ceramic (PDCs) routes predominantly concentrate on thermochemical modulation; specifically, the controlled pyrolysis temperature parameters governing their charge storage capabilities and electrochemical stability. Su et al. [44] synthesized SiCN materials by pyrolyzing polyethylenimine at 600–1500 °C. Compared to samples obtained at 600–800 °C (containing organic groups) and 1400–1500 °C (containing crystalline SiC), those produced between 1000–1300 °C, which incorporate free carbon, exhibit significantly higher charge–discharge capacities as anodes. The diminished capacity observed at ≥1500 °C is attributed to the formation of electrochemically inactive SiC phases [45,46]. Reinold et al. [47,48] highlighted that selecting the optimal calcination temperature involves balancing its impact on hysteresis, rate capability, charge transfer resistance, stability, and specific capacity. Their investigation into polyphenylvinylsilicon carbide imide-derived SiCN anodes revealed that heating to 1300 °C reduces hydrogen content, markedly decreasing voltage-capacity hysteresis. Concurrently, the ordering of free carbon at this temperature leads to lower specific capacity. However, elevated temperatures also improve charge transfer resistance and enhance SEI (solid electrolyte interface) stability through the ceramic matrix.

Notably, the initial coulombic efficiency remains a significant challenge for carbon-rich SiCN anodes in lithium-ion batteries, showing little dependence on pyrolysis temperature. Similar to SiOC anodes, highly conductive materials such as carbon nanotubes (CNTs) [49,50] and graphene [51] can be introduced into the SiCN matrix to improve its conductivity. Moreover, the addition of high-capacity materials like Si can further enhance the capacity. For instance, Zhang et al. [50] studied the electrochemical performance of multi-walled carbon nanotube–SiCN composite materials. Using a simple ultrasound-assisted method combined with high-temperature pyrolysis to synthesize the samples, the CNTs were evenly distributed within the SiCN ceramic matrix. This distribution maintained structural integrity during the polymer-to-ceramic transformation and formed a strong bond with the SiCN ceramic matrix. The resulting composite material showed superior rate performance compared to the original SiCN powder, CNTs, and graphite, achieving a capacity of 222.7 mAh∙g^−1^ at a current density of 2000 mA g^−1^.

Additionally, Bhandavat et al. [23] found that boron could modify the nanostructure of SiCN, improving its chemical stability and electronic conductivity. Furthermore, the cycling stability of the battery increased as “microporous activity” improved, which aligns with previous studies on SiOC ceramics [52]. Feng et al. found that it was recognized that increasing the porosity of SiCN negative electrode materials can improve the capacity and cycling performance of lithium-ion batteries. Therefore, a porous SiCN-HF electrode material was obtained by etching SiCN materials with HF aqueous solution. After conducting charge discharge cycling performance tests on it, it was found that compared to dense SiCN, its cycling stability was significantly improved. The principle is that it is easy for lithium-ions to be intercalated and detached after treatment, thereby alleviating the capacity attenuation caused by volume changes. A new type of material using porous SiCN-HF material as the negative electrode material for lithium batteries has been achieved [53]. Similarly, the laboratory subjected NaOH aqueous solution to the same etching treatment, and in the same charge discharge cycle performance test, compared to SiCN material, the discharge specific capacity and cycle stability were significantly improved. The principle is that the nano diameter pores formed after treatment facilitate the insertion and removal of lithium-ions [53].

Feng’s team [49] synthesized a composite material of SiCN and graphite (SiCN-graphite) by pyrolyzing a mixture of amorphous SiCN derived from polysilicone ethylenediamine and graphite powder. The SiCN-graphite material, used as the negative electrode active material for lithium-ion batteries, demonstrates outstanding electrochemical performance. Following the same charge–discharge cycle test as in the previous experiment, the SiCN-C anode demonstrated high initial and stable specific discharge capacities. Under the same charging and discharging conditions, both values were significantly higher than those of pure polymer-derived SiCN and C. Additionally, the SiCN-C negative electrode demonstrates high-rate charge–discharge capability, meeting the standards for commercial negative electrode materials. The fewer voids and cracks at the interface of SiCN-C particles, the more conducive they are to electron transfer, thereby enhancing capacity and cycle stability.

## 4. Other Si-Based Anode Materials

In addition to the widely studied SiOC and SiCN, researchers have also conducted some exploratory work on the application of polymer precursor ceramics such as SiBCN and SiOCN in the anode of lithium-ion batteries.

**Figure 9:**
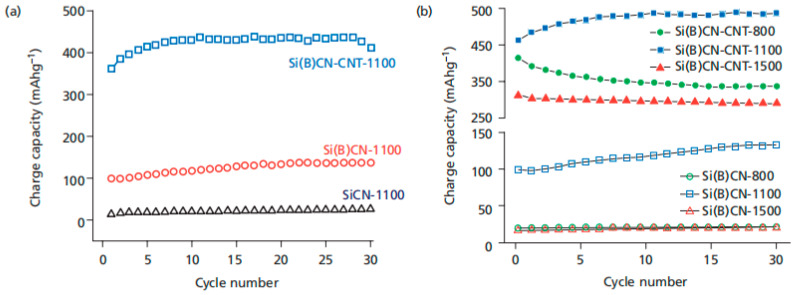
(**a**) Cycling performance of SiCN, Si(B)CN, and Si(B)CN-CNT composite anodes. (**b**) The best performance, characterized by the minimal loss in the first cycle and the highest charge capacity, was achieved by the specimen synthesized at approximately 1100 °C. Copied with permission from American Chemical Society [23].

SiBCN, polymer-derived quaternary silicoboron carbonitride (SiBCN) ceramics offer several advantages, including high strength, low density, excellent high-temperature stability, and strong resistance to oxidation and creep [54]. These properties make them ideal materials for high-temperature applications, such as fibers, bulk ceramics, composites, and membranes. Recently, SiBCN-based ceramics have attracted growing interest as anode materials for lithium-ion batteries. For instance, Bhandavat et al. investigated the electrochemical performance of SiBCN composites modified with carbon units (e.g., carbon nanotubes, graphene oxide) as anode materials for lithium batteries [17,18]. The results revealed that pure SiBCN ceramics exhibited a low discharge capacity of less than 101 mAh∙g^−1^, but the incorporation of carbon units significantly enhanced their electrochemical performance [54]. Studies on the solid-state mixing of MWCNT with SiCN ceramic particles have also yielded promising results. These composites demonstrated a 14% improvement over SiCN, with a reversible capacity of approximately 750 mAh∙g^−1^ in the first cycle and 400 mAh∙g^−1^ after the 30th cycle [55]. A recent study has shown that Si(B)CN-based anodes outperform SiCN anodes in both effective capacity and cycle life. As illustrated in Figure 9, the reversible capacity of polysilazane-based SiCN increased fourfold when doped with boron and CNT.

Idrees et al. [56] studied the effect of nitrogen-sulfur co-doped graphene (NSGs) sheets on the electrochemical performance of SiBCN ceramics as an anode material for lithium-ion batteries. The nanocomposite exhibited a reversible capacity of 785 mAh∙g^−1^ at a current density of 450 mA g^−1^ over 800 cycles, with an average capacity decay of 0.006% per cycle. Its high cycling stability can be attributed to the stacked graphene sheets of NSGs and the increase in disordered carbon, such as the rearrangement of −sp^2^ carbon chains and the formation of B(C)N domains in the PDCs.

In order to improve the performance of SiBCN, Wang et al. [57] inserted GN sheets into the ceramic network of polymer-derived SiBCN through liquid dispersion (SiBCN/GN-ld) and solid phase blending (SiBCN/GN-sp) at 1100 °C. Electrochemical measurements revealed that the SiBCN/GN-ld exhibited an impressive first-cycle discharge capacity of 844.2 mAh g^−1^ at a current density of 80 mA g^−1^, surpassing the performance of SiBCN/GN-sp, SiBCN, and GN anodes. The discharge capacity decreased to 347 mAh g^−1^ and remained stable in this range over 30 cycles. It shows that the GN layers played a crucial role in stabilizing the SiBCN matrix and mitigating the expansion of the material structure during charge–discharge cycles. The relevance between the electrochemical capacities of the SiBCN/GN composites and their compositions and structures suggest broad prospects to improve the electrochemical performance of these materials through molecular design and/or structural control.

Doping with heteroatoms (such as boron or nitrogen) through pyrolysis of heteroatom-containing precursors [19,20] and fabricating composites with other materials like carbon-based substances (e.g., CNTs, graphene), metals and metal compounds have also been explored to enhance the electrochemical properties of SiOC materials. Just like in the field of SiOCN, for example, Bekheet et al. [58] synthesized an Sn/N-doped silicon oxycarbide (SiOCN) nanocomposite by pyrolyzing a precursor of poly(vinyl)silazane and tin acetate. The introduction of metallic Sn nanoparticles facilitated charge transfer within the amorphous SiOCN matrix. Despite these improvements, the reversible capacity of SiOC anodes remains significantly below its theoretical value due to the low ICE. One promising strategy to enhance performance is the incorporation of metal compounds, which can mitigate irreversible reactions and promote better charge transport within the SiOC anode [59]. Zhao et al. [60] prepared spherical SiOCN materials by using an aldehyde-amine condensation and high-temperature pyrolysis method, and improved the first coulombic efficiency of the SiOCN/Li half-cell from 73.6% to 90.4% by employing a chemical prelithiation method using a simple immersion in Li-BP/2-MeTHF solution.

**Figure 10:**
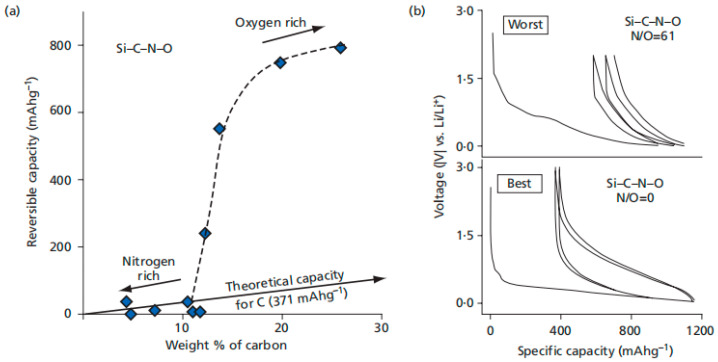
(**a**) The oxygen-rich SiCNO ceramic demonstrates a higher reversible capacity compared to the nitrogen-rich counterpart. (**b**) First charge/discharge cycles for SiCNO anodes prepared from nitrogen-rich (N/O = 61) and nitrogen-deficient (N/O = 0) specimens. Copied with permission from Elsevier [20].

Furthermore, Ahn et al. [20] explored the variation in ceramic precursor chemistry to synthesize a series of Si-C-N-O ceramics with different N/O ratios. The results indicate that SiOC exhibits superior performance compared to SiCN when processed under similar conditions (Figure 10). They concluded that the higher covalence of Si-N bonds localizes electron density, making them less effective for Li-ion binding compared to Si-O bonds. Additionally, they investigated the cyclic stability of SiOC as a function of pyrolysis temperature. Specimens pyrolyzed at 800 and 1000 °C retained nearly 100% of their capacity after 60 cycles, while those processed at 1200 and 1400 °C experienced a steady decline. Other studies have also reported improved electrochemical performance for SiOC specimens processed at 1100 and 1300 °C. And we can improve the performance through further investigation into Si-N and Si-O bonding and adjustment of pyrolysis temperature.

The practical evidence shows that utilizing SiOCN and SiBCN will become a promising approach to enhance battery performance.

## 5. Mechanism for Lithium-Ion Storage in Si-Based Anode Materials

### 5.1. Intercalation/De-Intercalation Process

**Figure 11:**
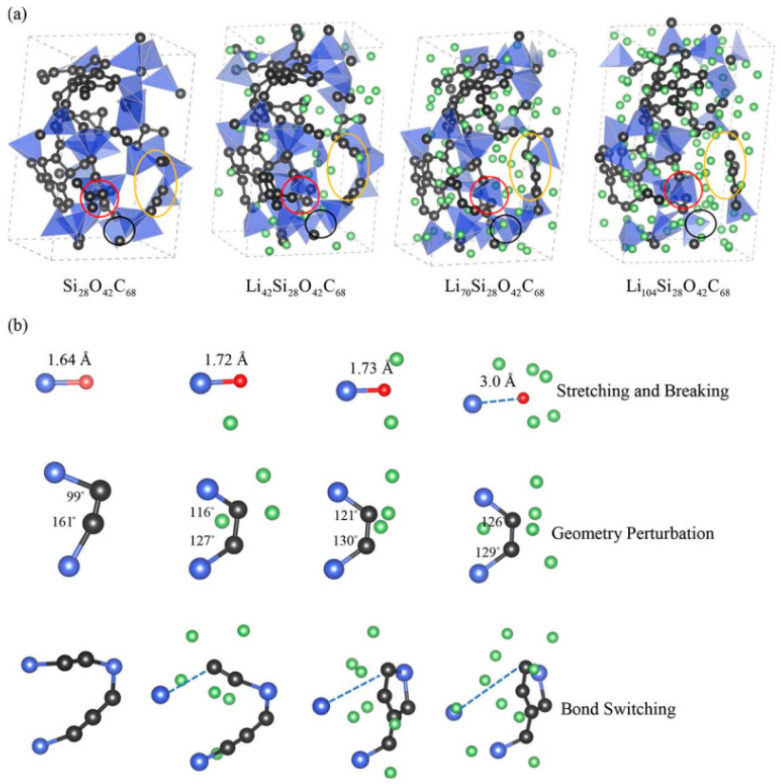
(**a**) Atomic configuration evolution within the SiOC matrix during lithiation reveals that the percolating carbon network backbone provides structural stabilization. Full lithiation induces a maximum cell volume expansion of 22%. (**b**) Local structural reorganization accompanying lithiation (depicted in (**a**)) involves: Si–O bond elongation and fracture, geometric distortion of Si–C–C–Si clusters, bond rearrangement within carbon rings. Atomic species are denoted: Si (purple), O (red), C (gray), Li (green). Copied with permission from American Chemical Society [62].

Regarding the lithium storage mechanism of SiOC, scientists have approached various angles of exploration. The intercalation and de-intercalation processes of lithium-ions are key to the electrochemical performance of polymer-derived ceramic anode materials. During intercalation, lithium-ions first diffuse through the electrolyte to the anode surface and then enter the material’s crystal lattice or amorphous structure (Figure 11). Liu [1] suggested that by comparing the capacity differentials of anode materials containing only Si, C, or O elements, the Si-O-C glass phase was confirmed to be the primary source of its reversible capacity. During intercalation, lithium-ions react with silicon, carbon, and oxygen elements in the material to form compounds such as Li_x_Si, Li_x_C, and Li_x_O, which constitute the basis for lithium-ion storage and release. The main lithium-intercalation platform of C/Si-O-C anode material corresponds to the interaction of the same lithium-ions in Si-O-C glass. The Si-O-C glass phase may have different lithium intercalation mechanisms.

During de-intercalation, lithium-ions are released from the material’s crystal lattice or amorphous structure and re-enter the electrolyte. Shi [13] noted that the structural evolution of different silicon units in SiOC ceramics during lithium-ion intercalation and de-intercalation significantly affects the reversibility of lithium-ions. Specifically, the silicon units in the Si-O-C glass phase undergo significant structural changes during lithium-ion intercalation and de-intercalation, directly impacting the material’s electrochemical performance and cycling stability [26].

Fukui et al. [61] used NMR for the first time to track the changes of Li before and after cycling in SiOC, demonstrating that SiOC contains three active sites where Li is stored: graphite interlayer or edge, SiOC phase, and micropores. After comparing the intensities of the three peaks, it was concluded that the graphite interlayer or edge is the main lithium storage active site in SiOC.

Additionally, Dragoljub Vrankovic and colleagues [19] used molecular dynamics simulations to study the lithium-ion intercalation and de-intercalation processes in porous silicon embedded in a ceramic matrix. They found that the diffusion and reaction behavior of lithium-ions in the porous structure significantly affects the material’s electrochemical performance. These studies indicate that optimizing the material’s microstructure and chemical composition is key to improving the electrochemical performance of polymer-derived ceramic anode materials. Moreover, free carbon is also important in lithium storage and the other roles of free carbon will be specifically discussed in Section 6.

### 5.2. Phase Transition Behavior

In polymer-derived ceramic anode materials, the intercalation and de-intercalation processes of lithium-ions induce significant phase transition behavior. Wen and colleagues [27] suggested that SiOC materials undergo a transition from SiC_4_ to SiO_4_ mixed bond configurations during lithium-ion intercalation, significantly impacting the material’s reversible capacity. Additionally, Liu ‘s doctoral thesis indicated that during the second cycle of charging in Si/Si-O-C anode materials, the de-intercalation process of nano-silicon and the Si-O-C glass phase overlaps but can be distinguished by the oxidation peaks in the potential range.

Graczyk-Zajac and colleagues pointed out that the carbon phase is the main lithium-ion storage site, with lithium-ions mainly accommodated at the edges and defect sites of graphene sheets, as well as at the interfaces of graphite nanocrystals. This phase transition behavior manifests as significant potential hysteresis phenomena during lithium-ion intercalation and de-intercalation, due to the kinetic and thermodynamic limitations of lithium-ion diffusion at the electrode and electrolyte surface.

In summary, polymer-derived ceramic anode materials exhibit complex phase transition behavior during lithium-ion storage, which not only affects the material’s electrochemical performance but also has significant implications for its cycling stability.

### 5.3. Structural Influence

The structure of polymer-derived ceramics (PDCs) anode materials has a significant influence on their lithium-ion storage performance. Firstly, the microstructure of SiOC materials undergoes notable changes during lithium-ion intercalation and de-intercalation. Wen and colleagues suggested that the mixed bond configurations (such as SiC_4_, SiC_3_O, SiC_2_O_2_, and SiCO_3_) in SiOC materials are the primary lithium intercalation sites, and their presence significantly enhances the material’s reversible capacity. Additionally, V.S. Pradeep [31] and colleagues noted that the combined effect of the amorphous silicon oxycarbide phase and free carbon phase in SiOC materials enables high carbon-content SiOC materials to exhibit excellent cycling stability and performance under high-rate charge and discharge conditions [63]. Secondly, the pore structure and particle size of the material also significantly impact its electrochemical performance. Murilo M Amaral [64] and colleagues indicated that the structure and porosity changes of PDCs materials during pyrolysis are influenced by temperature, precursor, and atmosphere, thereby affecting lithium-ion diffusion kinetics and battery energy efficiency. Dragoljub Vrankovic and colleagues suggested that the morphology of 3D Si/C/SiOC nanocomposites significantly impacts their electrochemical performance, and optimized nanostructures can significantly enhance lithium-ion storage capacity and cycling stability [65].

In summary, the structure of polymer-derived ceramic anode materials plays a crucial role in the mechanism of lithium-ion storage. By regulating the microstructure, porosity, and particle size of the material, its electrochemical performance and cycling stability can be significantly enhanced.

## 6. Role of Free Carbon in Si-Based Anode Materials

### 6.1. Improvement of Electrical Conductivity

In polymer-derived ceramics (PDCs) anode materials, the presence of free carbon plays a crucial role in improving electrical conductivity. Free carbon significantly improves the conductivity of the material by forming a conductive network, thereby improving the overall performance of lithium-ion batteries [66,67].

First, free carbon, as a conductive agent, is able to form a continuous conductive path in PDCs. This conductive network not only improves the conductivity of the material, but also enhances the transport efficiency of lithium-ions in the electrodes. Wu pointed out that by manipulating the pyrolysis parameters and microstructure of PDCs, the state of free carbon (such as content, crystallinity, and order) can be adjusted. The free carbon phase also helps to stabilize the amorphous structure of SiOC ceramics, resulting in enhanced chemical and thermal stability [68,69]. Based on the findings of Fukui, the reversible capacity observed during the initial discharge is attributed to the free carbon phase. The carbon phase in the non-etched silicon oxycarbide matrix can reversibly store nearly twice the amount of lithium compared to commercial graphite—723 mAh∙g^−1^ versus 371 mAh∙g^−1^, respectively. These values can be used to determine the x value in the LiC_x_ formula. For commercial carbon anodes, the x value is 6 (LiC_6_), whereas for silicon oxycarbide glasses, it is approximately 3. The enhanced reversible lithium storage capacity in the carbon phase formed in situ within the SiOC matrix may be due to the nanoscale dimensions of the carbon clusters, which allow lithium to be stored not only in the interstitial spaces of the graphite-like sp^2^-carbon structure but also at the edges of the graphene layers. After etching, the lithium storage capacity in the free carbon phase remains unchanged for the SiOC-10 composition, with an x value around 3 (LiC_3_). However, for the C-rich SiOC-200 sample, the x value increases from 3.7 in the non-etched sample to 5.4 after etching, approaching the lithium storage capacity of commercial graphite (x = 6) [70].

Increasing the free carbon content by modifying the Si-polymer with DVB improves the electrochemical performance of the resulting SiOC and SiCN ceramics, but only in the case of intrinsically carbon-poor preceramic polymers. The introduction of carbon into carbon-rich PDCs may induce structural changes in the ceramic matrix, which stabilizes the free carbon. However, if the carbon content exceeds a certain threshold, the stabilization effect may be lost [70].

In addition, free carbon can also be used as an active substance to store lithium-ions, increasing the reversible capacity of the material. Liu [1] pointed out that although the carbon content of C/Si-O-C anode materials does not always account for the majority of the composition, the contribution of free carbon in the lithium intercalation process still accounts for most of the reversible capacity of the whole system. This shows that free carbon not only plays an important role in electrical conductivity, but also contributes significantly to improving the lithium storage performance of materials.

### 6.2. Enhancement of Structural Stability

Free carbon plays a crucial role in polymer-derived ceramics (PDCs) anode materials, especially in improving structural stability and mitigating volume changes. Wu believes that free carbon improves the cycling stability of materials by providing the main host sites for lithium-ions, such as the edges, interstitial spaces, and defect sites of single-layer graphite [69]. In addition, the presence of free carbon helps to mitigate the volume change of lithium-ions during charging and discharging. Since lithium-ions will cause the volume expansion and contraction of the material during the intercalation and expulsion process, the high conductivity and flexibility of free carbon can effectively buffer this volume change, reduce the mechanical stress of the electrode material, and thus improve the cycling stability of the battery. Fox et al. [26] found that the presence of free carbon in 3D Si/C/SiOC nanocomposites significantly improves the electrochemical stability and cycle life of the materials.

Furthermore, the disordered nature of free carbon makes it exhibit excellent electrical conductivity during electrochemical processes, which is essential for maintaining the structural stability of PDCs anode materials. Kaspar et al. [69] pointed out that as the heat treatment temperature increases, the microstructure of free carbon tends to be ordered, which reduces the storage site of lithium-ions, but also enhances the structural stability of the material. At high temperatures, the presence of free carbon also contributes to the thermal stability of the material. Ren et al. [36] showed that although excess free carbon may lead to a decrease in thermal stability, the structural stability of the material is significantly enhanced in the presence of the right amount of free carbon. This enhancement is mainly attributed to the stable structure formed by free carbon at high temperatures. Wen et al. [27] found that the increase of free carbon can significantly improve the initial charge–discharge capacity and cycling stability of the materials by studying SiCN composites with different carbon content. They point out that free carbon not only provides more lithium-ion storage sites, but also significantly improves the electrochemical properties of the material through its excellent conductivity and structural stability.

### 6.3. Optimization of Electrochemical Performance

Free carbon in polymer-derived ceramics (PDCs) anode materials has a significant impact on their electrochemical properties. First, the presence of free carbon can significantly improve the cyclic stability and rate performance of the material. Reinold et al. [47] has shown that carbon-rich PDCs exhibit excellent stability during cycling, especially under high-temperature pyrolysis conditions, where the content and structure of free carbon have a direct impact on the electrochemical properties of the materials. In addition, Wu’s research [69] pointed out that the state of free carbon can be optimized by adjusting the pyrolysis temperature and microstructure, thereby significantly improving the electrochemical performance of PDCs anode materials. For example, by maintaining a high degree of disorder of free carbon, the cycle stability and rate performance of materials can be effectively improved.

Kaspar et al. suggested that an increase in free carbon content can significantly increase the carbon content of SiCN and SiOC ceramics, thereby improving the lithium-ion storage capacity and charge–discharge efficiency [63]. Their study found that as DVB was added, the free carbon content increased significantly, resulting in an increase in the reversible capacity of the material from 136 mAh∙g^−1^ to 574 mAh∙g^−1^ in the first cycle, while the coulombic efficiency increased by 10%. This view is also supported by Liu’s research, pointing out that free carbon can not only be used as an active material to store lithium-ions, but also as a conductive agent to improve the conductivity of C/Si-O-C systems [1]. His research shows that even at low free carbon content, the C/Si-O-C anode material still shows a reversible capacity much higher than the theoretical capacity of graphite, suggesting that the contribution of the relative reversible capacity of Si-O-C glass is also very important.

V.S. Pradeep et al. [31] found that the treatment temperature has a significant effect on the electrochemical properties of SiOC anode materials. For samples treated at 1000 °C, with an amorphous phase of 51 wt% and a free carbon of 49 wt%, it exhibits the highest reversible capacity and the best cycling stability. However, in high-carbon samples treated at 1300 °C, the reduction of free carbon leads to a decrease in reversible capacity and cycling stability. It shows that the free carbon is influenced by treatment temperature, and it is necessary to control treatment temperature to improve the performance of lithium-ion batteries.

Free carbon deeply influences the performance of materials. The performance of these materials is shown in Table 1 [58]:

In summary, the role of free carbon in PDCs anode materials is not only limited to providing lithium-ion storage sites, but more importantly, it effectively alleviates the volume change and improves the structural stability of the materials through its disordered structure and excellent electrical conductivity. These properties make free carbon a key factor in improving the performance of anode materials for PDCs. The role of free carbon in PDCs anode materials cannot be ignored. By optimizing the content and structure of free carbon, the electrochemical properties of the material can be significantly improved, including cycling stability and rate performance. These research results provide an important theoretical basis and experimental support for the future development of high-performance anode materials for lithium-ion batteries.

## 7. Challenges

Although PDCs demonstrate superior performance compared to conventional graphite anodes, their practical implementation faces critical challenges that necessitate systematic solutions.

(1) Capacity Limitation of Silicon-Glass Phases: The intrinsic properties of silicon-based glass phases inherently restrict capacity enhancement. While amorphous structures exhibit suboptimal long-term cycling stability and conductivity, strategic modifications—such as boron (B) and nitrogen (N) doping or composite design—can improve interfacial stability. Notably, the synergistic effects of multi-element doping (e.g., B-N co-doping) remain underexplored and warrant systematic investigation to unlock higher capacity retention.
(2) Low Initial Coulombic Efficiency (ICE): The persistently low ICE (50–70%) severely hinders commercial viability. To bridge this gap, targeted strategies including pre-lithiation techniques (chemical/electrochemical) and surface coatings must be optimized. The trade-offs between ICE improvement and long-term cycling stability require quantitative analysis to establish optimal processing parameters.
(3) Unresolved Interfacial Interactions: Compared to mainstream anode materials, the interactions between PDCs and other battery components (current collectors, binders, electrolytes) lack comprehensive understanding. Specifically, the correlation between particle size distribution and electrochemical performance (e.g., rate capability, SEI formation) remains ambiguous. The role of PDCs–electrolyte interphase dynamics in dictating cycling behavior needs mechanistic clarification.
(4) Complex Storage Mechanisms and Performance Metrics: The capacity storage mechanism involves intricate interfacial reactions and phase transformations, where performance degradation correlates strongly with structural evolution. Key gaps include absence of standardized evaluation protocols for quantifying capacity fade mechanisms, incomplete understanding of how microstructural features (e.g., free carbon domains, Si nanoclusters) evolve during cycling, and lack of consensus on performance benchmarks (e.g., acceptable ICE thresholds, capacity retention rates).

## 8. Perspectives

Future research should focus on the following key directions to advance the practical application of the polymer-derived Si-based anode materials:

(1) Multi-Component Synergistic Design and Molecular Engineering: Develop multi-component PDCs systems through precise molecular-level design to tailor silicon-carbon-heteroatom network structures. Establish structure–property relationships linking microstructure (porosity, interfacial phases) to lithium storage mechanisms (alloying/intercalation/conversion reactions).
(2) Advanced In Situ Characterization: Utilize advanced in situ platforms (e.g., in situ XRD/Raman) to dynamically resolve lithium-ion diffusion pathways within amorphous networks, track phase evolution during cycling, and reveal microscopic capacity fade mechanisms. This will provide theoretical guidance for structural optimization.
(3) Development of Green Scalable Synthesis: Explore low-energy, environmentally benign synthetic routes, prioritizing precursor selection and reaction condition optimization (e.g., low-temperature adaptations of hydrothermal/sol-gel methods), strategies balancing cost control with yield enhancement, bridging the technical gap between lab-scale synthesis and industrial production.
(4) Establishment of Standardized Testing Protocols: Develop unified electrochemical performance evaluation standards, including normalized data reporting formats (e.g., capacity retention rate calculation methods, rate capability testing procedures). Industry-wide adoption of these benchmarks will accelerate the translation from laboratory research to commercialization.

Concurrent progress across these domains will systematically address the current scientific challenges and engineering bottlenecks of PDCs, paving the way for next-generation high-energy-density lithium-ion batteries.

## 9. Conclusions

In summary, silicon-based polymer-derived ceramics (PDCs), including SiOC, SiCN, SiBCN, and SiOCN, etc., have emerged as promising anode materials for next-generation lithium-ion batteries (LIBs) due to their easy structural design and modulation, chemical stability, etc. These materials overcome the limitations of conventional graphite anodes, such as low theoretical capacity (372 mAh·g^−1^), and mitigate the severe volume expansion (>300%) associated with pure silicon anodes.

PDCs synthesized through precursor pyrolysis exhibit unique amorphous/nanocrystalline structures. The synergy between silicon-based glass phases and free carbon significantly boosts lithium-ion storage capacity and cycling stability: SiOC achieves >1300 mAh·g^−1^ reversible capacity through mixed Si-O-C bonding and free carbon networks, while SiCN’s nanochannels/active sites enable exceptional kinetics. Free carbon serves as a cornerstone for performance optimization, constructing conductive networks, buffering volume changes, and providing additional Li-storage sites via disordered carbon phases.

Crucially, PDCs’ microstructure and electrochemical properties are governed by pyrolysis temperature, atmosphere, and precursor design. Inert atmospheres retain disordered carbon to enhance capacity, whereas temperatures >1400 °C increase crystallinity at the expense of active sites. Incorporating conductive frameworks (e.g., CNTs, graphene) or metal nanoparticles (Sn, Si) further optimizes conductivity and mechanical stability, enhancing capacities and cycle life.

In conclusion, silicon-based polymer-derived ceramics (PDCs), with their design flexibility and tunable properties, hold great potential as high-energy-density anode materials for lithium-ion batteries (LIBs). Their inherent compatibility with solid-state electrolytes—notably in mitigating interfacial resistance and suppressing lithium dendrite formation—positions PDCs as particularly promising candidates for next-generation solid-state batteries. Through multi-scale structural optimization and interdisciplinary innovation, PDCs are poised to play a pivotal role in advancing energy storage technologies beyond traditional LIBs.

## Figures and Tables

**Table 1:** The effect of free carbon on the performance of lithium-ion batteries. The free carbon content, first-cycle reversible capacity (Crev), irreversible capacity (Cirr) and coulombic efficiency (η) as well as cycling current and if available capacity retention upon continuous cycling are listed (n.d. = not determined) [62].

Samples	Free Carbon [wt%]	*C_rev_* [mAh∙g^−1^]	*C_irr_* [mAh∙g^−1^]	*η* [%]	Cycling Current [mA g^−1^]	Capacity Retention	Ref.
SiO_1.5_C_3.9_	44.3	640	340	65	14.8	n.d.	[30]
SiO_0.51_C_7.78_	65.2	608	259	70	32.7	95% after 40 cycles	[61]
SiO_0.85_C_1.99_	25.9	794	370	68	100	n.d.	[71]
SiO_0.90_C_4.40_	48.5	568	330	63	18	cycling stable	[72]
SiO_0.98_C_2.47_	32.0	605	325	65	18	cycling stable	[31]
SiO_1.59_C_3.36_	43	600	680	47	360	cycling stable	[73]
SiO_1.18_C_5.52_	54.2	504.3	287.1	63.7	37	68.8% after 60 cycles	[66]
SiO_0.95_C_3.72_	43.6	535.9	335.8	61.5	37	56.0% after 60 cycles	[66]
SiO_1.01_C_2.93_	36.8	434.3	273.8	61.3	37	58.7% after 60 cycles	[66]
SiO_0.93_C_2.26_	29.5	501.4	302.7	62.3	37	47.3% after 60 cycles	[66]
SiO_0.87_C_1.62_	20.6	682.5	495.8	57.9	37	13.5% after 60 cycles	[66]
SiO_1.00_C_1.05_	11.6	706.1	375.5	65.3	37	5.2% after 60 cycles	[66]
SiO_1.40_C_0.70_	8.1	500.7	754.6	39. 9	37	1.5% after 60 cycles	[66]
SiC_5.35_N_0.98_O_0.19_	57.04	383	172	69	18	cycling stable	[46]
SiC_3.70_N_0.69_O_0.62_	46.02	241	291	45	18	cycling stable	[45]
SiO_0.06_C_1.54_N_0.74_	23.2	69	67	50.6	18.6	127.5% after 114 cycles	[74]
SiO_0.05_C_2.22_N_0.84_	33.4	278	199	58.3	18.6	112.9% after 114 cycles	[74]
SiO_0.10_C_4.04_N_0.69_	49.3	374	227	60.5	18.6	115.9% after 114 cycles	[74]
SiC_3.9_O_0.1_N_0.8_	48	703	375	65	18	89% after 134 cycles	[52]
SiC_10.59_O_1.56_N_0.21_	69.1	570	367	61	18	cycling stable	[75]
SiC_3.7_O_0.1_N_1.3_H_0.9_	48.1	674	525	56	18.6	68% after 134 cycles	[47]
SiC_5.3_O_0.3_N_1.2_H_0.2_	56.0	282	224	56	18.6	109% after 134 cycles	[47]
SiOC-phenyl	37.5	793	394	67	100	cycling stable	[76]
SiOC-propyl	14.3	687	660	51	100	cycling stable	[76]

## Data Availability

No new data were created or analyzed in this study. Data sharing is not applicable to this article.
